# Balancing Panels: Lets Call on Women Speakers

**DOI:** 10.1002/jgh3.70178

**Published:** 2025-05-13

**Authors:** Swapna Gayam, Hamsika Moparty, Manya Pala, Shivangi Kothari

**Affiliations:** ^1^ Section of Gastroenterology and Hepatology West Virginia University Morgantown West Virginia USA; ^2^ Department of Internal Medicine Brooklyn Hospital Center New York City New York USA; ^3^ Department of Internal Medicine Southern Regional Medical Center Riverdale Georgia USA; ^4^ Division of Gastroenterology and Hepatology University of Rochester Medical Center Rochester New York USA

**Keywords:** conference speaking, gender equity, women leadership, women speakers, women visibility

## Abstract

**Background:**

Gender inequality in medical conference speaking is glaring, women are vastly under‐represented. Lack of gender diversity in leadership spills into speaking opportunities at conferences, leading to a self‐propagating cycle of gender inequality. Breaking this cycle is vital to closing the gap, since speaking at conferences is a key element of academic advancement.

**Aims:**

This manuscript aims to highlight the paucity of women speakers at medical conferences despite adequate expertise, the importance of bringing balance to the stage and ways to improve visibility of women speakers.

**Methods:**

Extensive search was conducted for articles on pubmed, google scholar, Science Direct, WVU Library database using manels.

**Conclusion:**

Increased visibility of women on stage encourages women attendees and trainees to pursue leadership and speaking opportunities, which is key to “closing the gender gap” for future generations. Gender balanced panels on stage is an important aspect of “Diversity and Inclusion,” which will motivate and encourage other women workforce to follow suit.

## 1 Current State

The current state of gender diversity in medical conferences is far from satisfactory. Studies have shown that women are significantly underrepresented as speakers at conferences, with only about 30% of speakers being women [1]. This lack of representation is due to lack of women in medical leadership positions. Despite an increase in women enrolling in medical schools and becoming professionally active physicians, the representation of women in medical leadership positions remains sparse [2, 3, 4, 5, 6, 7]. In 2005, approximately 16 000 students graduated from U.S. medical schools, with women comprising 45% (7200 women). By 2015, this increased to 18 700 graduates, with women representing 47% (8800 women). Despite these overall gains, female representation in competitive specialties like gastroenterology, cardiology, orthopedic surgery, and general surgery remains uneven. According to the ACGME Data Resource Book, women in surgery increased from 27% in 2007–2008 to 46.7% in 2022–2023. Gastroenterology rose from 28% to 40.1%, while cardiology grew from 18% to 27.3%. However, orthopedic surgery lags behind, with female representation climbing only from 12% to 20.3% in the same period, indicating the need for further action [8, 9] (Table 1). This disparity could be attributed to reasons like lack of guidance and mentorship, lack of institutional support, and lack of accommodation for maternity needs and childcare responsibilities [4, 10]. This scarcity of female role models in leadership positions perpetuates the gender stereotypical construction of leadership, as highlighted by Sealy and Singh in their book “Leadership Perspectives” [11]. The paucity of women medical professionals in leadership positions translates to the underrepresentation of women as conference speakers because conference organizers prefer to invite academic leaders to speak. In turn, conference speaking being an important criterion for academic faculty promotion leads to a self‐perpetuating cycle of gender inequality, where women are continually underrepresented, leading to fewer speaking opportunities and hampered academic advancement [12, 13, 14, 15]. Conversely, conference speaking boosts visibility and promotes academic success, leading to increased future speaking opportunities, thereby inducing a successful feedback loop [16].

**TABLE 1:** Percentage of active female residents by specialty and subspecialty from ACGME data resource book [9].

Year	Surgery (%)	Orthopedic surgery (%)	Gastroenterology (%)	Cardiology (%)
2007–2008	27.0	12.0	28.0	18.0
2010–2011	32.0	12.0	30.0	21.0
2015–2016	36.1	14.0	33.6	21.0
2020–2021	43.9	16.7	36.9	24.1
2022–2023	46.7	20.3	40.1	27.3

Arora et al. conducted one of the largest studies till date on gender inequity of medical specialty conferences. In their international, multi‐specialty cross‐sectional analysis across 27 medical specialties from March 2017 to November 2018, they noted 30.1% women speakers with a median of 30.5% at each conference [1]. One of the specialties examined by Arora et al. in their multinational study was GI. In the US, the study identified 24.8% women speakers at GI conferences, 41.4% manels (panels that have all males are known as “manels”) and only 4.3% all female panels. This is very similar to global numbers in Australasia, Canada, Europe and UK, a mean of 26% women speakers in GI [1].

US Gastroenterology societies have also conducted internal reviews of women faculty in society programs. The American Society of Gastrointestinal Endoscopy (ASGE) has shown an increasing trend of women course faculty between 2009 and 2014 [16]. A more recent review by Jansson‐Knodell and Bhavsar‐Burke et al. studied the visibility of women at the American College of Gastroenterology (ACG) Annual meetings and demonstrated an increasing trend over a 10‐year time period, 2010–2019. Rising trends were noted in the percentage of women serving as Post Graduate course (11.7%–30.5%) and Annual Scientific Meeting (14.1%–45.4%) faculty and the proportion of overall women speakers (11%–44%) [17]. Despite a positive trend, there is still under‐representation of women speakers at medical conferences.

## 2 The Need to Change the Current State

Gender diversity has been found to enhance decision making and improve outcomes in any discussion, as it brings fresh perspectives, broader experience, and new ideas to the table. According to a recent article by Larson E, gender diverse teams out‐perform “all male teams” [18].

Increasing women speakers at medical conferences is a good step towards promoting gender diversity. Conferences provide an ideal platform to showcase equity, diversity, and inclusivity [12]. When women trainees and colleagues see other women speakers, it inspires and motivates them to participate in the conference, thereby maximizing its impact [15, 16]. The absence of women on stage perpetuates their absence at the event. When most presenters are men, it promotes a sense of exclusion for women attendees, resulting in fewer women choosing to speak up or even attend, thereby limiting the diversity of views and ideas in discussions [12]. In fact, this makes women feel silenced and might even lead to women quitting their academic careers [19]. It has been noted that, as the percentage of female speakers increases, so does the percentage of female attendees, and when there are more women on stage, women attendees feel more welcome at the conference [20, 21].

## 3 Limitations

One significant limitation to addressing the lack of gender diversity in medical conferences is the lack of awareness and understanding of the problem. Conference organizers may not realize the extent of the problem or may not be aware of ways to address it. Additionally, there may be a lack of accountability or incentive for conference organizers to improve gender diversity, leading to slow or no progress.

Another limitation is the challenge of finding qualified and available women speakers. With the underrepresentation of women in medical leadership positions, finding women speakers with the necessary qualifications and availability can be difficult, leading to a reliance on the same speakers and perpetuating the cycle of gender inequality.

Finally, there may be resistance to change, with some individuals and organizations hesitant to act to address gender inequality due to fear of potential backlash or uncertainty about the best approach.

While there are certainly limitations to achieving gender diversity in medical conferences, it is essential to continue to work toward this goal, as increased diversity leads to better decision‐making and outcomes, and greater inclusivity and equity.

## 4 Solutions

To achieve gender equality and diversity, it is imperative to increase the representation of women in medical leadership and conferences simultaneously. Conference organizers can take steps to expand their pool of academic leadership to include more women, provide more opportunities for women to speak, and promote gender diversity and inclusivity in conference programming. Additionally, ongoing efforts are necessary to monitor the gender representation of speakers at conferences, provide training and mentorship for women speakers to improve their speaking skills, and sustain the proposed model.

One potential solution to increase women speakers at medical conferences is to establish mentorship and leadership programs that support and promote women in the field. Organizations such as ACG‐Women in GI, Women in Endoscopy (WIE), Women in Cardiology (WIC), Women in Innovations (WIN), Women as One, The Association of Women Surgeons (AWS) and American Academy of Orthopedic Surgeons (AAOS) have been instrumental in supporting female physicians through their mentorship, leadership programs, and advocacy for research funding. While these organizations have made significant contributions, it is clear that we need more of these initiatives to promote gender equity, support career advancement, and advocate for systemic changes to improve the professional environment for women in these fields.

Another solution could be to implement blind review processes for conference presentations, where reviewers do not have access to the names or gender of the presenters. This approach can help reduce bias in the selection process of future speakers and ensure that the most qualified and relevant speakers are chosen.

Furthermore, conference organizers can also prioritize diversity and inclusivity in their selection process by actively seeking out and inviting women and underrepresented minorities as speakers. This can be achieved by establishing partnerships with organizations that focus on promoting diversity in medicine and reaching out to potential speakers who may not be well known but have valuable perspectives and expertise to share.

Penfold et al. provided practical steps that should be taken by conference organizers, attendees and women speakers to ensure gender equity among speakers. This includes actively seeking out women with relevant knowledge and experience and encouraging them with speaking opportunities, mentoring and coaching for inexperienced speakers and being accommodative to women speakers' needs like child care [12]. A list of “Ten simple rules” to achieve conference gender balance, as compiled by Martin JL, includes collecting and reporting speaker data, building speaker databases, and developing policies to address gender disparities [22].

Women speaker databases and websites like learn.g2.com help conference organizers find potential women speakers and prevent manels [23]. Women Speakers in Healthcare (WSH), a grassroots initiative, established England's largest database of women healthcare professionals interested in speaking at conferences [12].

Other training programs include campaigns like #PresentHER and #CentHERStage speaker bootcamps, which encourage and coach women speakers at conferences [23, 24].

Another solution is to increase the number of women conference chairs, directors, and conveners. Research has shown that having a woman chair or co‐chair can lead to a significant increase in the number of women speakers at a conference, as seen at the Annual Society of American Gastrointestinal and Endoscopic Surgeons (SAGES) meeting from 2009 to 2018. Importantly, this increase in women speakers did not come at the expense of male speakers, demonstrating that diversity is not a zero‐sum game. Women conveners often prioritize diversity when organizing sessions, which can lead to more inclusive and representative speaker lineups [14].

It may not be enough to simply #CurbManels; instead, we should strive for balanced panels. One effective approach is to adopt gender equity targets, as demonstrated by the National Health Service (UK) [12]. Ultimately, sustaining the proposed model for increasing the representation of women speakers at medical conferences will require ongoing commitment and action from conference organizers, academic institutions, and the broader medical community. It will require sustained efforts from not only women but also men, who can be allies by providing inclusion, promotion, nomination, sponsorship, mentorship, and true #HeForShe support [25, 26]. Initiatives like #NominateHer and #HeForShe provide essential platforms that encourage both emerging and experienced female faculty members to pursue leadership and speaking opportunities. By supporting these efforts, institutions can help dismantle barriers that have historically limited women's advancement in academic medicine while fostering mentorship from male allies and mentors. The #NominateHer initiative specifically aims to reduce unconscious bias by encouraging both men and women to nominate qualified female colleagues for leadership roles. Similarly, #HeForShe programs advocate for male mentors to support the professional development of female physicians, making gender equity a shared responsibility. These programs help close the gender gap in conference representation, ensuring female physicians are recognized for their contributions, thereby promoting diversity and inclusion in academic communities.

Additionally, leading medical institutions are adopting practical strategies to promote work‐life balance, which is essential for retaining skilled professionals. For example, Stanford Medicine offers flexible scheduling, pregnancy disability leave, and parental bonding leave to support new mothers and employees with caregiving responsibilities. Massachusetts General Hospital supports this effort with a generous parental leave policy, offering up to 12 weeks for both parents to ensure smooth training continuity without hindering career progress. The University of Michigan Health further enhances these efforts by offering lactation rooms and dedicated time for breastfeeding, allowing new mothers to balance their personal and professional responsibilities. Together, these efforts by academic institutions and professional organizations contribute to a more inclusive and supportive work environment, empowering women to balance family responsibilities while progressing in their careers.

## 5 Conclusion

Despite efforts to address gender inequality in medical leadership and speaking opportunities at conferences, there is still much work to be done.

“If she can see it, she can be it [27]. We owe it to future generations to show that gender should not limit one's potential. Being mindful of diversity and inclusion when planning any course, conference, or initiative can be a starting point for developing more women role models for the next generation.”

## Conflicts of Interest

The authors declare no conflicts of interest.

## References

[1] A. Arora , Y. Kaur , F. Dossa , R. Nisenbaum , D. Little , and N. N. Baxter , “Proportion of Female Speakers at Academic Medical Conferences Across Multiple Specialties and Regions,” JAMA Network Open 3, no. 9 (2020): e2018127. 32986107 10.1001/jamanetworkopen.2020.18127 PMC7522699

[2] Accessed April 10, 2023, https://www.aamc.org/media/9716/download?attachment.

[3] Association of American Medical Colleges , “Medical School Enrollment More Diverse in 2021. Diversity and inclusion. Medical Education,” 2021.

[4] https://www.aamc.org/data‐reports/faculty‐institutions/interactive‐data/data‐reports/faculty‐institutions/interactive‐data/2021‐us‐medical‐school‐faculty.

[5] J. Boylan , J. Dacre , and H. Gordon , “Addressing Women's Under‐Representation in Medical Leadership,” Lancet 393, no. 10171 (2019): e14. 30739700 10.1016/S0140-6736(18)32110-X

[6] D. S. Jamorabo , R. Chen , H. Gurm , et al., “Women Remain Underrepresented in Leadership Positions in Academic Gastroenterology Throughout the United States,” Annals of Gastroenterology 34, no. 3 (2021): 316–322. 33948055 10.20524/aog.2021.0597 PMC8079863

[7] S. J. Diamond , C. R. Thomas , S. Desai , et al., “Gender Differences in Publication Productivity, Academic Rank and Career Duration Among US Gastroenterology Faculty,” Academic Medicine 91, no. 8 (2016): 1158–1163. 27144993 10.1097/ACM.0000000000001219

[8] “American Board of Internal Medicine Resident & Fellow WorkForce data. Home About ABIM Statistics & Data,” accessed March 21, 2022.

[9] ACGME Data Reource Book, https://www.acgme.org/about/publications‐and‐resources/graduate‐medical‐education‐data‐resource‐book/.

[10] D. M. Blumenthal , A. R. Olenski , R. W. Yeh , et al., “Sex Differences in Faculty Rank Among Academic Cardiologists in the United States,” Circulation 135, no. 6 (2017): 506–517. 28153987 10.1161/CIRCULATIONAHA.116.023520 PMC5308800

[11] R. H. V. Sealy and V. Singh , “The Importance of Role Models in the Development of Leaders' Professional Identities,” in Leadership Perspectives (Springer, 2008), 208–222.

[12] G. McLachlan , R. Penfold , K. Knight , et al., “Women Speakers in Healthcare—Surfing the Tide of Change, The Bmjopinion, July 17,” 2019.

[13] S. M. Ruzycki , S. Fletcher , M. Earp , et al., “Trends in the Proportion of Female Speakers at Medical Conferences in the United States and Canada, 2007 to 2017,” JAMA Network Open 2, no. 4 (2019): e192103. 30977853 10.1001/jamanetworkopen.2019.2103 PMC6481599

[14] T. C. Dumitra , M. Trepanier , L. Lee , et al., “Gender Distribution of Speakers on Panels at the Society of American Gastrointestinal and Endoscopic Surgeons (SAGES) Annual Meeting,” Surgical Endoscopy 34, no. 9 (2020): 4140–4147. 31605219 10.1007/s00464-019-07182-2

[15] A. Larson , K. M. Sharkey , J. A. Poorman , et al., “Represeantation of Women Among Invited Speakers at Medical Specialty Conferences,” Journal of Women's Health (Larchmont) 29, no. 4 (2020): 550–560. 10.1089/jwh.2019.7723 31687866

[16] B. K. Enestvedt , R. S. DeVivo , C. M. Schmitt , and A. H. Calderwood , “Increase in Female Faculty in American Society for Gastrointestinal Endoscopy‐Sponsored Programming Over Time,” Gastrointestinal Endoscopy 87, no. 4 (2018): 952–955, 10.1016/j.gie.2017.09.031. 28987546

[17] C. L. Jansson‐Knodell , I. Bhavsar‐Burke , S. Gayam , S. Kothari , and A. S. Oxentenko , “Visibility of Women at the American College of Gastroenterology Annual Meetings Over Time,” American Journal of Gastroenterology 116, no. 10 (2021): 2149–2151. 34114569 10.14309/ajg.0000000000001345

[18] E. Larson , “Infographic: Diversity+Inclusion= Better Decision Making at Work, Cloverpop.com, September 19,” 2017.

[19] J. Biggs , P. H. Hawley , and M. Biernat , “The Academic Conference as a Chilly Climate for Women: Effects of Gender Representation on Experiences of Sexism, Coping Responses, and Career Intentions,” Sex Roles 78 (2018): 394–408.

[20] Accessed March 22, 2022, www.womenspeakersinhealthcare.co.uk.

[21] “More Women On Stage at Conferences,” accessed March 21, 2022, Stefaniegrieser.com.

[22] J. L. Martin , “Ten Simple Rules to Achieve Conference Speaker Gender Balance,” PLoS Computational Biology 10, no. 11 (2014): e1003903. 25411977 10.1371/journal.pcbi.1003903 PMC4238945

[23] “Learn.g2.com/women‐speakers,” accessed March 21, 2022.

[24] “Unbounce.com > call to action > # CentHERstage: How to promote Gender Diversity,” accessed March 21, 2022.

[25] NHS Confederation , “Men as Allies,” 2019, accessed March 31, 2022; accessed March 20, 2022, www.nhsconfed.org/sites/default/files/media/Men‐as‐allies.pdf.

[26] M. Bilal , S. Balzora , M. B. Pochapin , and A. S. Oxentenko , “The Need for Allyship in Achieving Gender Equity in Gastroenterology,” American Journal of Gastroenterology 116, no. 12 (2021): 2321–2323. 34665160 10.14309/ajg.0000000000001508

[27] “Geena Davis Institute on Gender in Media‐See Jane,” accessed March 31, 2022, https://seejane.org.

